# C-reactive protein and white blood cell count are adverse prognostic markers for patients with advanced cancer on parenteral nutrition in a palliative care unit setting: A retrospective cohort study

**DOI:** 10.1177/02692163211073939

**Published:** 2022-02-20

**Authors:** Markus Kieler, Paul Kössler, Matija Milovic, Elias Meyer, Kristína Križanová, Lea Kum, Alexander Friedrich, Eva Masel, Raimund Bauer, Matthias Unseld

**Affiliations:** 1 Center for Physiology and Pharmacology, Institute for Vascular Biology, Medical University Vienna, Vienna, Austria; 2 Department of Medicine I, Division of Palliative Medicine, Medical University of Vienna, Vienna, Austria; 3 Center for Medical Statistics, Informatics, and Intelligent Systems, Medical University of Vienna, Vienna, Austria; 4 Center for Pathobiochemistry and Genetics, Institute of Medical Chemistry and Pathobiochemistry, Medical University of Vienna, Vienna, Austria

**Keywords:** Parenteral nutrition, C-reactive protein, white blood cell count, cancer, biomarkers, palliative care

## Abstract

**Background:**

Parenteral nutrition is controversial in patients with advanced cancer. Nevertheless, this treatment is common practice near the end of life.

**Aim:**

We aimed to identify factors which were associated with the outcome of patients on parenteral nutrition at an academic tertiary palliative care unit.

**Design:**

In this retrospective cohort study patients were assigned to two groups according to parenteral nutrition treatment. Inferential statistics were used to assess whether the dynamics of laboratory variables over 2 weeks of parenteral nutrition were associated with survival.

**Setting/Participants:**

Patients admitted to the Department of Palliative Medicine at the Medical University of Vienna between 2016 and 2018 were included in this study.

**Results:**

Of 443 patients, 113 patients received parenteral nutrition. Patients had a lower body mass index, lower levels of bilirubin, γ-glutamyltransferase, alkaline phosphatase, and were of younger age compared to patients which did not receive parenteral nutrition. No difference in survival as measured from admission to death was found when comparing the two groups. Levels for γ-glutamyltransferase, alkaline phosphatase, and C-reactive protein significantly increased during 2 weeks of parenteral nutrition. Among patients with parenteral nutrition, an increase in C-reactive protein or white blood cell count levels was associated with lower survival.

**Conclusion:**

Patients who responded with an increase of C-reactive protein or white blood cell count during 2 weeks after reinitiation or start of parenteral nutrition had a worse survival. Our findings might support clinicians and patients in their decision to forgo parenteral nutrition in a palliative care setting.

**What is already known about the topic**

- Parenteral nutrition is common practice in the Western world, parts of Asia, and Latin America for patients with advanced cancers near the end of life, although there is a lack of high quality data which show benefit for this treatment.
- Inflammatory markers like the C-reactive protein/albumin ratio have been validated for assessing the mortality risk in unselected patient groups with parenteral nutrition or cancer patients without parenteral nutrition.

**What this paper adds**

- In the palliative care unit setting patients with advanced cancers on parenteral nutrition who have rising C-reactive protein- and white blood cell count levels have an increased risk of death.

**Implications for practice**

- C-reactive protein- and white blood cell count levels might be used for the selection of patients who likely do not benefit from parenteral nutrition. These findings could help to limit futile treatment near the end of life.

## Background

About 50%–80% of patients with advanced cancers experience cancer-related cachexia and malnutrition which poses a huge clinical and economic burden as these conditions are associated with increased morbidity and mortality.^1–6^ Although there is clear data which show that nutritional treatments via the oral and enteral route can have positive effects in cachectic cancer patients,^7–13^ the level of evidence decreases on when to use parenteral nutrition in patients especially if these patients do not have severe gastrointestinal insufficiency.^14–16^ Tobberup et al.^17^ concluded in their recent systemic review about parenteral nutrition for patients with advanced cancer that the nutritional status of advanced cancer patients might improve but with little gains in quality of life and that parenteral nutrition was not superior to fluid administration for terminal patients in regards of survival. The authors also again pointed out the weak level of evidence for current parenteral nutrition treatment in these patients. Nevertheless, parenteral nutrition in patients with advanced cancer is a frequent practice with reported rates up to 53%.^18^ A cross-sectional study among the palliative care research network in Sweden showed that parenteral nutrition was predominately initiated when patients presented with weight and appetite loss but still had oral intake.^19^ This underscores the inconsistencies between recommendations in clinical guidelines that are based on current evidence and actual practices.

Owing to these circumstances, health care professionals often face uncertainties in their decision regarding provision of parenteral nutrition or forgoing it in patients with advanced cancer in the course of their disease. In this respect, the identification of easily measurable and readily available blood variables that can be used to estimate the prognosis of these patients would help in advance care planning. Studies aiming at identifying such markers in a well-defined cohort of patients with advanced cancer have not been reported. Therefore, the aim of this study was to examine if there is an association between the dynamics of routinely measured lab variables and the clinical outcome of patients on parenteral nutrition at a tertiary palliative care unit. Our results show that among this group, patients with increasing levels of C-reactive protein- or white blood cell count had an inferior survival. This might have implications for future clinical practice as it could help to identify patients who are unlikely to benefit from parenteral nutrition and support clinicians and patients in their decision to forgo unnecessary treatment at the end of life.

## Materials and methods

### Study design

This study represents a retrospective data analysis of patients who have been treated at the Division of Palliative Medicine of the Medical University of Vienna between January 2016 and September 2018.

### Population and setting

Data of 443 patients were retrieved from the electronic database of the Medical University of Vienna.

### Participant sampling approach

The levels of laboratory variables from two time points were queried. These were before the application of parenteral nutrition (time point 0 [T0]) and in the second week after initiation of parenteral nutrition (T2). Patients who did not receive parenteral nutrition were used as a control group. Levels of laboratory variables during the first 2 weeks of admission were used concordantly.

### Parenteral nutrition regimens

Parenteral nutrition was administered according to the individual needs of the patients as assessed by the dietologist of the Division of Palliative Medicine. The formula consisted of NuTRIflex^®^ Omega special (with electrolytes) and a well-established mixture of Soluvit, Vitalipid, and Trace.

### Statistical analysis

Descriptive statistics of all variables of interest were calculated. Logistic regression models were used to assess differences in body mass index, age, and sex in relation to C-reactive protein—white blood cell count—and liver function tests levels between patients who received parenteral nutrition and those that did not. Since there are outliers in the blood levels, sandwich estimates for the covariance matrix were used. Paired *t*-tests were utilized to determine the dynamics of C-reactive protein—white blood cell count—and liver function tests levels from T0 to T2 for the patients who received parenteral nutrition. Cox-regressions were computed to assess the association of parameters of interest with overall survival. For assessing the influence of parenteral nutrition application on overall survival, the variables parenteral nutrition, sex, age, and body mass index or a Kaplan-Meier estimator were used. For assessing the influence of the variables dynamics on overall survival the difference between the two points was used as variables in the univariate cox regressions. A multivariate cox regression analysis was used for the variables that were statistically significant in the univariate model to correct for the occurrence of systemic infections. This was assessed by screening the medical histories for clinical symptoms of infections (e.g. onset of fever, cough, diarrhea, etc.) and abnormalities in routinely performed chest X-rays and dipstick analysis in the first 2 weeks after admission to the palliative care unit. *P*-values < 0.05 were considered statistically significant. As *p*-values serve only descriptive purposes no multiplicity corrections were applied. To minimize a potential selection bias all patients who have been treated during the study period were included in the study and assigned to either the group with parenteral nutrition or without.

### Ethical considerations

The present study was conducted in accordance with the Declaration of Helsinki (World Medical Association, 2013). Ethical approval was gained by the Ethics Committee of the Medical University of Vienna (2019-02-19, EK Nr: 2185/2018).

## Results

### Patients’ characteristics

This study included data of 245 female and 198 male patients who were hospitalized during the observation period at the palliative care unit of the Medical University of Vienna. Table 1 describes patients’ characteristics. Parenteral nutrition was administered to 113 patients. Of these 113 patients, 34 patients had received parenteral nutrition already before admission and 79 patients were parenteral nutrition naïve. The two groups of patients with or without parenteral nutrition mainly differed in age, body mass index, and primary tumor origin.

**Table 1.** Patients’ characteristics.

No of patients (*n*, %)	443 (100)	
Male (*n*, %)	198 (45)	
Female (*n*, %)	245 (55)	
	*Patients with PN*	*Patients without PN*
*No of patients*	113 (100)	330 (100)
Mean age (SD, years)	60.12 (12.96)	64.71 (12.08)
Mean BMI (SD)	20.02 (3.36)	24.63 (15.02)
<18	45 (40)	47 (14)
18–25	55 (49)	173 (52)
25–30	6 (5)	37 (12)
>30	2 (2)	34 (10)
Missing	5 (4)	39 (12)
Metastasis	100 (88)	280 (85)
No metastasis	13 (12)	50 (15)
Tumor origin		
Colorectal cancer	13 (12)	41 (12)
Pancreas	24 (21)	29 (9)
Gastric/Esoophagus	9 (8)	8 (2)
HCC/CCC	5 (4)	11 (3)
Lung	11 (10)	74 (22)
Breast	6 (5)	47 (14)
Head and neck	7 (6)	17 (5)
Reproductive organs	11 (10)	16 (5)
RCC/Urothelial	2 (2)	15 (5)
Sarcoma	7 (6)	18 (6)
Blood	6 (5)	17 (5)
NET	3 (3)	4 (1)
Brain	3 (3)	10 (3)
Other	6 (5)	23 (7)

Patients’ characteristics; PN: parenteral nutrition; HCC: hepatocellular carcinoma; CCC: cholangiocarcinoma; RCC: renal cell carcinoma; NET: neuroendocrine tumor.

### Association of age, body mass index, and bilirubin at baseline with parenteral nutrition

Patients with younger age, lower body mass index, and bilirubin were more likely to receive parenteral nutrition. The other variables sex, liver function tests, C-reactive protein, and white blood cell count levels showed no association with parenteral nutrition treatment. Results are shown in Table 2.

**Table 2.** Comparison of baseline variables between patients with parenteral nutrition or without.

	OR	Lower CI	Upper CI	*p*-Value
Male	0			
Female	0.771	0.443	1.341	0.357
**Age**	**0.96**	**0.937**	**0.983**	**<0.001**
**BMI**	**0.809**	**0.757**	**0.865**	**<0.001**
**Bilirubin**	**0.769**	**0.669**	**0.884**	**<0.001**
GOT	0.992	0.982	1.002	0.106
GPT	1.003	1	1.007	0.082
γGT	1	0.999	1.001	0.627
AP	1.001	1	1.003	0.14
CRP	1.013	0.981	1.047	0.428
WBC	0.975	0.936	1.016	0.231

GPT: glutamic-pyruvic transaminase; γGT: γ-glutamyltransferase; AP: alkaline phosphatase; CRP: C-reactive protein; WBC: white blood cell count.

Logistic regression of baseline variables and administration of parenteral nutrition: all patients (*n* = 371); glutamic oxaloacetic transaminase.

### Dynamics of laboratory variables

The difference of the laboratory variables over time from T0 to T2 was analyzed. Patients with parenteral nutrition had rising levels of γ-glutamyltransferase, alkaline phosphatase, and C-reactive protein. For the group of patients without parenteral nutrition a significant increase in white blood cell count levels was detected within the first 2 weeks after admission. Results are shown in Table 3.

**Table 3.** Dynamics of laboratory variables.

Patients without parenteral nutrition (*n* = 330)						
	Mean (T[0])	Mean (T[2])	Mean Diff	Lower CI	Upper CI	*p*-Value
Bilirubin (mg/dl)	1.781	1.593	−0.189	−0.638	0.261	*0.409*
GOT (U/L)	63.432	101.312	37.88	−9.928	5.689	*0.120*
GPT (U/L)	35.627	37.699	2.073	−8.026	12.171	*0.686*
γGT (U/L)	285.24	313.682	28.443	−11.756	68.642	*0.164*
AP (U/L)	232.508	245.915	13.407	−8.328	35.143	*0.225*
CRP (mg/dl)	8.180	8.598	0.418	−0.796	1.631	*0.498*
**WBC (G/L)**	**10.105**	**11.551**	**1.446**	**0.520**	**2.372**	***0.002***
Patients with parenteral nutrition (*n* = 113)						
Bilirubin (mg/dl)	0.787	0.981	0.194	−0.292	0.681	0.429
GOT (U/L)	39.55	40.438	0.888	−10.616	12.391	0.878
GPT (U/L)	36.244	35.679	−0.564	−12.142	11.013	0.923
** γGT (U/L)**	**239.825**	**316.663**	**76.838**	**10.281**	**143.394**	**0.024**
** AP (U/L)**	**206.533**	**298**	**91.467**	**49.572**	**133.362**	**<0.001**
** CRP (mg/dl)**	**8.387**	**11.652**	**3.264**	**1.011**	**5.518**	**0.005**
WBC (G/L)	10.298	10.999	0.701	−1.224	2.626	0.47

Logistic regression of the changes between T0 and T2 for the respective groups.

GOT: glutamic oxaloacetic transaminase; GPT: glutamic-pyruvic transaminase; γGT: γ-glutamyltransferase; AP: alkaline phosphatase; CRP: C-reactive protein; WBC: white blood cell count.

### Survival times

**Figure 1.**
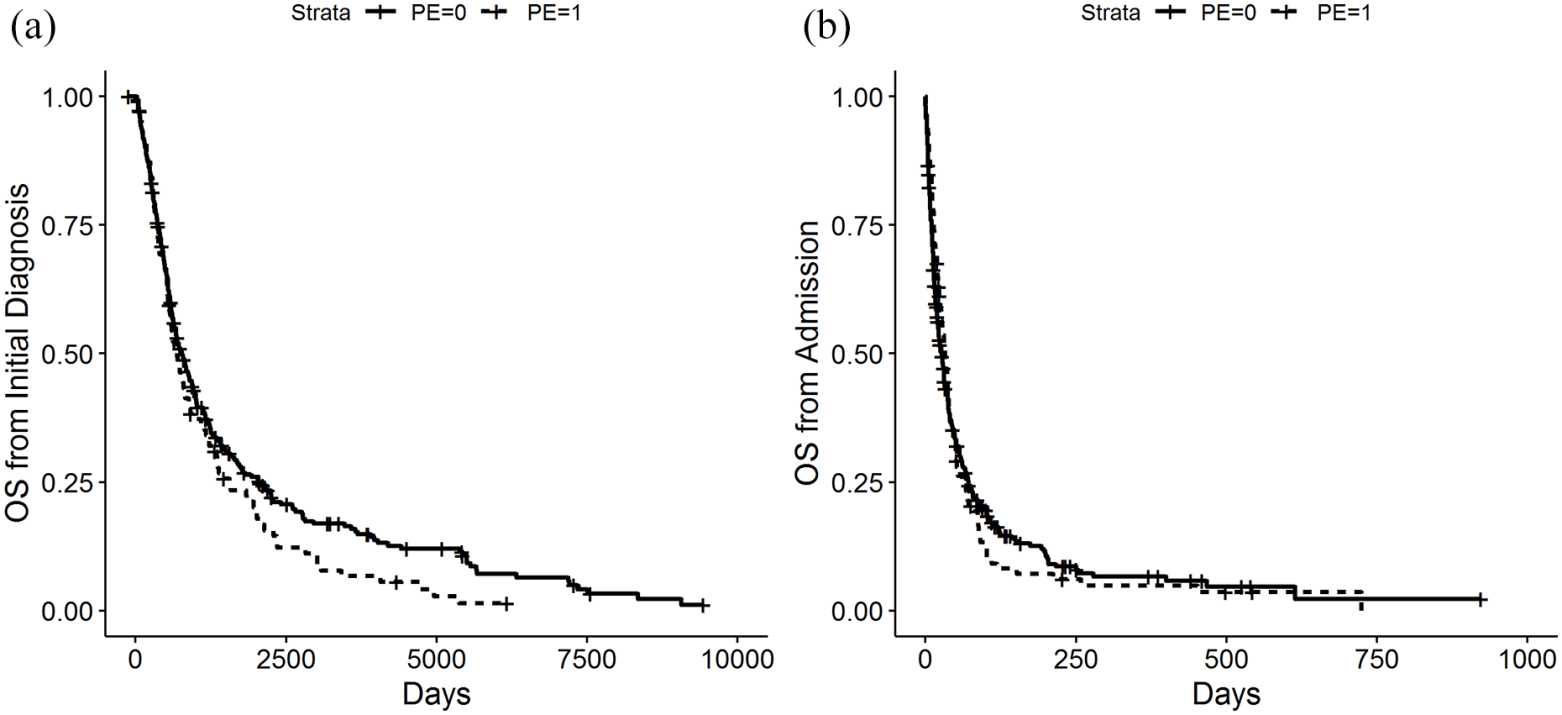
(a) Survival probability measured from the time of the initial diagnosis and (b) Survival probability measured from the time of the admission to the palliative care unit.

No significant difference in overall survival was found when patients were stratified according to parenteral nutrition status. Survival was calculated from the date of first diagnosis (HR 1.172, CI [0.901, 1.523], *p*-value = 0.237) and from the date of the admission to the palliative care unit (HR 0.947, CI [0.737, 1.218], *p*-value 0.673). The median survival for patients with parenteral nutrition was 33 days from the date of admission and 22.8 months from date of initial diagnosis. For the group of patients without parenteral nutrition the median survival times were 27 days and 25.2 months. Kaplan–Meier curves are presented in Figure 1.

### Clinical outcome and association with patients’ characteristics and laboratory variables at baseline

In the group of patients with parenteral nutrition no association between sex, age, body mass index, or baseline levels of laboratory variables with overall survival as calculated from admission to the palliative care unit was found. Results are shown in Table 4.

**Table 4.** Influence of patients’ characteristics and baseline variables on the survival of patients with parenteral nutrition.

	Hazard Ratio	Lower CI	Upper CI	*p*-Value
Age	0.996	0.982	1.011	0.593
Sex (female)	0.856	0.579	1.267	0.437
BMI	1.052	0.997	1.11	0.063
Bilirubin (T0)	1.129	0.976	1.306	0.102
GOT (T0)	1.001	0.998	1.004	0.443
GPT (T0)	1	0.996	1.003	0.829
γGT (T0)	1	1	1.001	0.111
AP (T0)	1.001	1	1.001	0.151
CRP (T0)	1.016	0.992	1.042	0.192
WBC (T0)	1.02	0.998	1.043	0.077

Cox-regression: BMI: Body Mass Index; GOT: glutamic oxaloacetic transaminase; GPT: glutamic-pyruvic transaminase; γGT: γ-glutamyltransferase; AP: alkaline phosphatase; CRP: C-reactive protein; WBC: white blood cell count.

### Clinical outcome and association with the dynamics of the laboratory variables

In the group of patients without parenteral nutrition rising C-reactive protein levels in the course of 2 weeks of admission were associated with shorter survival. For patients on parenteral nutrition, additionally to C-reactive protein levels, also rising white blood cell count levels indicated shorter survival. These associations were still significant when correcting for the occurrence of systemic infections as assessed with chest X-rays, dipstick test, or onset of clinical symptoms like fever. Results are shown in Table 5.

**Table 5.** Influence of dynamics of levels of laboratory variables (T0–T2) on survival.

Patients without parenteral nutrition				
	Hazard Ratio	Lower CI	Upper CI	*p*-Value
Univariate model				
Bilirubin (T(2)-T(0))	1.028	0.972	1.088	0.336
** GOT (T(2)-T(0))**	**1.001**	**1**	**1.001**	**0.006**
GPT (T(2)-T(0))	1.004	1	1.008	0.063
γGT (T(2)-T(0))	1	0.999	1.001	0.952
AP (T(2)-T(0))	1	0.999	1.002	0.661
** CRP (T(2)-T(0))**	**1.037**	**1.001**	**1.075**	**0.047**
WBC (T(2)-T(0))	1.019	0.98	1.059	0.348
Patients with parenteral nutrition				
Univariate model				
Bilirubin (T(2)-T(0))	1.034	0.904	1.181	0.628
GOT (T(2)-T(0))	1.002	0.997	1.008	0.447
GPT (T(2)-T(0))	1.003	0.998	1.008	0.256
γGT (T(2)-T(0))	1	0.999	1.001	0.949
AP (T(2)-T(0))	1.001	0.999	1.002	0.379
** CRP (T(2)-T(0))**	**1.026**	**1.005**	**1.048**	**0.017**
** WBC (T(2)-T(0))**	**1.038**	**1.003**	**1.075**	**0.035**
Multivariable model				
** CRP (T(2)-T(0))**	**1.027**	**1.006**	**1.049**	**0.013**
Chest X-ray	1.777	0.992	3.181	0.053
** CRP (T(2)-T(0))**	**1.028**	**1.006**	**1.05**	**0.012**
Dipstick test	2.16	0.898	5.197	0.086
** CRP (T(2)-T(0))**	**1.027**	**1.006**	**1.049**	**0.012**
Clinical symptoms of infection	1.35	0.837	2.178	0.218
** WBC (T(2)-T(0))**	**1.039**	**1.002**	**1.077**	**0.039**
Chest X-ray	1.651	0.919	2.967	0.094
** WBC (T(2)-T(0))**	**1.039**	**1.002**	**1.077**	**0.039**
Dipstick test	2.354	0.966	5.736	0.059
** WBC (T(2)-T(0))**	**1.042**	**1.006**	**1.08**	**0.021**
Clinical symptoms of infection	1.425	0.874	2.323	0.156

GOT: glutamic oxaloacetic transaminase; GPT: glutamic-pyruvic transaminase; γGT: γ-glutamyltransferase; AP: alkaline phosphatase; CRP: C-reactive protein; WBC: white blood cell count.

Univariate and multivariable cox-regression analysis.

## Discussion

### Main findings

In this retrospective study the dynamics of routinely assessed laboratory variables over a period of 2 weeks from patients with advanced cancers who received parenteral nutrition were assessed in relation to their survival. Increasing levels of C-reactive protein or white blood cell count were associated with shorter survival. These two markers might help in advance care planning and the decision whether to use or forgo parenteral nutrition in a palliative care setting.

### Discussion of main findings in relation to previous research about parenteral nutrition and prognostic markers

Currently, there is one study that evaluated different prognostic scores combining C-reactive protein with albumin or prealbumin in an all comer group of 460 patients with parenteral nutrition.^20^ Although the patient cohort and design of this study is not comparable to ours this study showed that C-reactive protein/albumin and C-reactive protein/prealbumin ratio correlates with the survival of patients on parenteral nutrition. In another study the combination of C-reactive protein levels of above 10 mg/L and serum-albumin levels of below 30 g/L was assessed in relation to the survival of patients with advanced cancer near the end of life.^21^ Around 85% of patients fulfilled these laboratory criteria in the last 30 days of their life. The authors concluded that C-reactive protein in the absence of clinical signs of infection might be used as an indicator of short remaining lifetime. Also other factors with prognostic value like the Karnofsky performance status or cholinesterase levels have been described.^22–24^ Our findings add to the current evidence that C-reactive protein- and white blood cell count levels are prognostic markers for the specific group of patients with advanced cancer on parenteral nutrition.

### Ethical and legal frameworks of parenteral nutrition for patients with advanced cancer: Considerations in relation to the results of this study

Provision of adequate fluid and nutrients is basic care and perceived as a fundamental human right.^25^ On the other hand parenteral and enteral feeding is defined as a medical treatment. Therefore, the legal principles which apply to parenteral nutrition are the same which apply to all other medical treatments like medication or ventilation.

The majority of guidelines from medical societies in the Western world endorse that the decision to provide parenteral nutrition should be based on the four principles of bioethics; autonomy, beneficence, nonmaleficence, and justice.^26–29^ Patients can refuse all medical interventions at any time and it is the duty of the treating physician to inform them about this right. Conversely, respecting the autonomy does not mean that a patient should obtain every treatment which he demands. When determining if a medical intervention is justified the remaining three principles of bioethics must be acknowledged. A key consideration for beneficence is the definition of a clear and realistic therapeutic goal. An evident medical indication for parenteral nutrition in a patient with incurable cancer is if the patient is expected to die sooner from starvation than from tumor progression. The principle of nonmaleficence involves not providing a treatment where the burdens and risks outweigh any potential benefits. Referring to these principles the European Society for Clinical Nutrition and Metabolism mentions in its consensus statement from 2016 that “the continued medical justification for artificial nutrition must be reviewed at regular intervals, determined in accordance with the patient’s condition.”^26^ This is particularly important when patients are expected to have a prognosis with a short survival period because the reasons which initially led to decision to start parenteral nutrition might no longer exist. Generally, there is a clear preference for forgoing parenteral nutrition for patients near the terminal phase. Guidelines from the European Society for Clinical Nutrition and Metabolism suggest that if a patient has a prognosis of less than 2 months this treatment is deemed inappropriate.^30^ However, accurately estimating the remaining lifetime of patients with cancer is often not possible and the gradual sometimes slow progression to the terminal phase complicates this further. A relevant question concerning parenteral nutrition and patients with a very limited prognosis is also if the decision to forgo this treatment is associated with distress and suffering for the patient. In this context it should be noted that patients in the terminal phase of their disease rarely experience hunger.^31^ A sensation of thirst or dry mouth can be effectively relieved with mouth care and small amounts of liquids. Conversely, in this phase the continued provision of parenteral nutrition and hydration might lead to increased symptom burden for example from nausea, fluid overload, hyperglycemia, incontinence, and infections. There is a lack of evidence that parenteral nutrition provides clear benefit for these patients.^32^ Increasing levels of C-reactive protein or white blood cell count could indicate that parenteral nutrition might be futile medical care as the patient approaches the end of life and should encourage the treating physician to reconsider the medical, ethical, and legal justification of this treatment.

### Expectations concerning the role of parenteral nutrition near the end of life

The individual expectations and attitudes towards parenteral nutrition at the end of life can also cause concerns among patients and their relatives. In a survey about decisions concerning artificial nutrition and hydration in a palliative care setting around 40% of patients and relatives opted for forgoing artificial nutrition at the end of life.^33^ Relatives were however more reluctant when deciding on behalf of the patient. This can create conflicts between the obligations of the treating physician and the expectations especially in situations involving patients who lack decision-making capacity. Furthermore, preferences of patients require an accurate understanding of their prognosis. This includes not only the stage, type of cancer, and comorbidities but is also dependent on the individual perception of the deciding person and the cultural and spiritual background of the patient and the clinician.^34^ In this respect, our results can also be helpful when clinicians discuss with patients and their relatives whether to forgo parenteral nutrition near the end of life in cases where this treatment is deemed futile.

### Ethnical and regional differences of end-of-life care with a focus on parenteral nutrition: Implications of local practices on the results of this study

There are local variations in end-of-life care that are based on differences in laws, traditions, rules, religious beliefs, and ethical directions which all have an impact on medical practices. In the majority of European countries, the United States, Canada, and Australia legal and ethical rules place patient autonomy at the center of the decision making process. This implicates that the patient should be informed about the prognosis of the disease and that the care provider needs to obtain consent from the patient before forgoing treatment. The patient centered autonomy approach of the Western World is in contrast to that of Asian countries like China, Japan, and Taiwan where family centered decision-making is the rule.^35^ Patients often have a low level of autonomy in these countries and treatment decisions are mostly delegated to members of their family.^36,37^ The so-called principle of “filial piety” often leads to the decision to not forgo life-sustaining treatment which can also include maintaining artificial nutrition until the end of life.^38^ A recent systemic review about attitudes and preferences toward palliative care in Latin America points out that patients would like to be informed about their prognosis and that they tend to prefer shared or active over passive decision-making.^39^ However, it seems that caregivers are hesitant to discuss this with their patients. In light of these local and ethnical differences the results of this study mainly apply to practices where there is a strong focus on the patient’s autonomy and where physicians support advance care planning together with the patient.

### Limitations

Limitations of the study include its retrospective design which limits the ability to conclude on the causal relationship between implementation of parenteral nutrition and an elevation of inflammatory markers. The assumption of a significant result is based upon the cut-off *p*-value of 0.05 and is not corrected for multiple testing. Furthermore, we did not exclude patients with liver metastases or existing liver conditions which might influence our results regarding liver function tests.

## Conclusion

Our study explored the relationship between routinely assessed laboratory variables in the course of parenteral nutrition treatment of patients with advanced cancer. Increasing C-reactive protein- and white blood cell count levels were associated with an increased risk for death. These prognostic markers can be helpful to raise awareness for futile medical care as patients might approach the end of life where the burdens and potential risks of artificial nutrition most likely outweigh any benefits.
